# Significance of Sodium Bisulfate (SBS) Tempering in Reducing the *Escherichia coli* O121 and O26 Load of Wheat and Its Effects on Wheat Flour Quality

**DOI:** 10.3390/foods10071479

**Published:** 2021-06-25

**Authors:** Jared Rivera, Aiswariya Deliephan, Janak Dhakal, Charles Gregory Aldrich, Kaliramesh Siliveru

**Affiliations:** 1 Department of Grain Science and Industry, Kansas State University, Manhattan, KS 66506, USA; jdrivera07@ksu.edu (J.R.); aish@ksu.edu (A.D.); aldrich4@ksu.edu (C.G.A.); 2 Department of Food Science and Technology, Virginia Tech, Blacksburg, VA 24060, USA; janakdhakal_vet@hotmail.com

**Keywords:** shiga toxin-producing *E. coli*, sodium bisulfate, wheat tempering

## Abstract

The occurrence of recalls involving pathogenic *Escherichia coli*-contaminated wheat flours show the need for incorporating antimicrobial interventions in wheat milling. The objectives of this study were to assess the efficacy of sodium bisulfate (SBS) tempering in reducing *E. coli* O121 (ATCC 2219) and O26 (ATCC 2196) wheat load and to evaluate the impact of effective (≥3.0 log reductions) SBS treatments on wheat flour quality. Wheat grains were inoculated with *E. coli* (~6 log CFU/g) and tempered (17% moisture, 24 h) using the following SBS concentrations (%wheat basis): 0, 0.5, 0.75, 1.0, 1.25, and 1.5% SBS. Reductions in *E. coli* O121 and O26 wheat load at different time intervals (0.5, 2, 6, 12, 18, and 24 h) during tempering were evaluated. The addition of SBS during tempering resulted in *E. coli* (O121 and O26) log reductions of 2.0 (0.5% SBS) to >4.0 logs (1.5% SBS) (*p* ≤ 0.05). SBS tempering (1.25 and 1.5% SBS) produced acidic wheat flours (pH = 4.51–4.60) but had comparable wheat flour properties in terms of composition, dough, and bread-making properties relative to the control (0% SBS). SBS tempering reduced the *E. coli* O121 and O26 load of wheat after tempering with minimal effects on wheat flour quality.

## 1. Introduction

Wheat is a raw agricultural commodity that is susceptible to microbial contamination from various sources (e.g., soil, water, insects, etc.) in its supply chain. The presence of enteric microorganisms such as *E. coli* and *Salmonella* was also reported [1]. Due to the physical nature of the milling process, the wheat microflora is redistributed into the mill fractions produced. Furthermore, antimicrobial intervention steps are currently not included in the milling process. These factors increase the risk of foodborne pathogens contaminating the wheat flour produced if they are initially present in the milled wheat grains [2].

Wheat flours were historically perceived as a microbiologically safe ingredient due to their low water activity (a_w_) [3]. However, pathogenic microorganisms such as Shiga toxin-producing *E. coli* (STEC) were reported to survive under dry conditions through various mechanisms such as the accumulation of osmoprotectants and reverting to a dormant state [4]. This capability of pathogens reinforces the risk of wheat flours being a potential route for foodborne illness in humans. Furthermore, once these foodborne pathogens contaminate wheat flours, they were reported to survive for significant periods of time (≥nine months) under ambient temperature storage conditions [5].

Wheat flours are often viewed as a raw ingredient, and products using wheat flours usually undergo effective thermal processing steps (e.g., baking, extrusion) before consumption. However, foodborne illness outbreaks persist because of the consumption of raw or undercooked dough or batters by consumers. The risk for wheat flours as a vehicle for foodborne illnesses was exhibited by the increased occurrence of outbreaks and recalls linked to wheat flour-based products. An outbreak related to STEC (O157)-contaminated wheat-based products occurred in 2009 wherein a prepackaged cookie dough caused 77 illnesses (35 hospitalizations, eight HUS cases) in the US [6]. Another multi-state outbreak linked to STEC (O121 and O26)-contaminated wheat flour occurred in 2016 wherein 63 cases across 24 US states were recorded [7]. These outbreaks typically resulted in the recall of significant amounts of affected products. In addition, the occurrence of recalls involving STEC-contaminated wheat flours has increased compared to previous years, with 13 recalls occurring in 2019 [8]. The occurrence of these events presents a significant risk of economic loss for wheat flour manufacturers.

These events highlight the need for improving wheat flour food safety through the inclusion of food safety interventions in the milling process. Non-thermal processes that were previously reported include ozone, chlorine, and cold plasma treatment of wheat grains before milling [9,10]. These methods were reported to be effective in reducing the microbial load of the wheat grains prior to milling. However, these processes involve major modifications in the milling process and also present added worker safety risks due to chemical and dust explosion hazards. These factors limit the practicality and cost-effectiveness of these non-thermal methods, which limited their integration into commercial-scale wheat milling operations [3]. Thermal processing of wheat flours was also established. However, the adoption of this intervention is limited by its high cost of operation associated with the added equipment and energy cost as significantly longer heating times are required for wheat flours due to its poor thermal conductivity.

The inclusion of antimicrobial agents such as acids (e.g., lactic, acetic, and citric acid) and saline solutions in the tempering step were reported to be effective in improving the microbial quality of wheat [11]. The inclusion of these acids in the tempering water were also reported to have minimal impacts on wheat flour functionality. Hence, modifications in the tempering step could provide a viable route for incorporating food safety interventions in the milling process.

Sodium bisulfate (SBS) is a GRAS (Generally Recognized as Safe) acidulant commonly used in food manufacturing operations as an acidifier and anti-browning agent. It was hypothesized in this study that the increased acidity brought by adding SBS to the tempering water could be an effective intervention step to reduce STEC contamination levels in wheat after tempering. Therefore, the objectives of this study were to evaluate the efficacy of SBS tempering in reducing *E. coli* O121 and O26 loads of wheat after tempering and to assess the impact of effective SBS tempering concentrations (≥3 log reductions) on wheat flour quality.

## 2. Materials and Methods

### 2.1. Materials

The hard red winter (HRW) wheat grains used for this study were obtained from Indigo Agriculture (Boston, MA, USA). The microbial counts (log CFU/g) obtained for the wheat samples used in this study are aerobic counts–4.6, coliforms–4.1, *E. coli*–negative (after enrichment), yeast and molds–not detected (<1 log CFU/g), and *Enterobacteriaceae*–3.5. The sodium bisulfate (SBS) powder used was provided by Jones-Hamilton Co. (Walbridge, OH, USA).

### 2.2. Inoculum Preparation

*Escherichia coli* O121 (ATCC 2219) and O26 (ATCC 2196) cultures were obtained from the American Type Culture Collection (ATCC) (Manassas, VA, USA). Cultures were maintained in tryptic soy broth (TSB): glycerol (7:3) solution at −80 °C. Thawed cultures were streaked in tryptic soy agar (TSA) plates and incubated (37 °C; 24 h). A well-isolated colony of each *E. coli* strain from the streak plate was individually inoculated in TSB (10 mL) and incubated (37 °C, 24 h). The cells were harvested by centrifugation (2795× *g*, 10 min; Sorvall X1R, Waltham, MA, USA) and were inoculated in sterile TSB (800 mL) and incubated (24 h; 37 °C). Cells from the incubated TSB (800 mL) were harvested by centrifugation (2795× *g*; 10 min) and re-suspended in sterile 0.1% peptone water (tempering experiments) or sterile TSB (MIC determination). This resulted in an inoculum concentration of ~9 log CFU/mL.

### 2.3. Minimum Inhibitory Concentration (MIC) Assay

The minimum inhibitory concentration (MIC) of SBS in TSB was conducted using the broth microdilution method based on standardized protocols of the Clinical and Laboratory Standards Institute [12]. *E. coli* (O121 or O26) in TSB (∼6 logs CFU/mL) was added (100 µL) to each well containing 100 µL of decreasing concentrations (three wells/concentration) of SBS solutions yielding a final volume of 200 µL per well. Positive (*E. coli* inoculum only) and negative (TSB only) controls were maintained. The microdilution plate was incubated (37 °C, 24 h), and MIC was determined as the lowest concentration that inhibited visible *E. coli* growth after incubation. The MIC study was replicated twice.

### 2.4. Wheat Preparation, Inoculation, and Application of Tempering Solutions

Wheat grains were placed in metal containers and sterilized by autoclaving (121 °C, 15 min) to reduce background microflora. Sterile wheats were cooled at ambient room temperature (24 h) prior to the tempering experiments. The moisture content (% wet basis) of sterile wheats was determined based on American Society of Agricultural Engineers (ASAE) 352.2 method [13]. Sterile wheat (200 g) was tempered to 17% moisture with the amount of tempering solution required (16 mL) calculated based on the initial moisture content (10.4%). SBS solutions were prepared by diluting a 50% (*w*/*v*) SBS stock solution to the required concentrations that would yield the target SBS tempering concentrations of 0.5, 0.75, 1.0, 1.25, and 1.5% SBS (wheat basis) when applied. These concentrations correspond to the following SBS solution concentrations (% *w*/*v*): 13.4, 20.3, 26.9, 33.7, and 40.5%. The SBS solutions were prepared fresh prior to the tempering experiments. The required SBS solution concentrations (% *w*/*v*) required to achieve the SBS tempering concentrations (% wheat basis) were calculated using the equation below:

%SBS (%*w*/*v*) = ([(%SBS wheat basis) × (weight _tempered wheat_)])/(volume _tempering solution_) (1)

Sterile wheat (200 g) was spray-inoculated with *E. coli* O121 or O26 inoculum (~9 log CFU/mL, 8 mL) using a standard bottle sprayer nozzle giving inoculation levels of ~6 log CFU/g. Inoculated wheats were rested for 30 min to allow cell attachment. Sprayers were washed with 70% ethanol and rinsed with sterile water before each inoculation procedure. The prepared SBS treatments (8 mL) were spray-applied into the inoculated wheat giving final SBS tempering concentrations of 0.5, 0.75, 1.0, 1.25, and 1.5% SBS. Wheat containers were sealed, and samples were shaken vigorously (5 min) after the inoculation, and SBS applications and were left to temper for 24 h (22–25 °C). Positive (inoculated wheat + 0% SBS) and negative controls (sterile wheat + sterile water) were also maintained. Inoculation and tempering of wheat kernels were done under aseptic conditions inside a Biosafety Cabinet.

### 2.5. Microbiological Analysis

For the first objective, treated wheats were aseptically sampled (25 g) at the following time intervals: 0.5, 2, 6, 12, 18, and 24 h upon application of the tempering treatments; 25 g of wheat was mixed with 225 mL buffered peptone water (BPW) in stomacher bags (VWR, Radnor, PA, USA) and homogenized (2 min) using a stomacher (Seward, Islandia, NY, USA). Serial decimal dilutions in 0.1% peptone water and appropriate dilutions were spread plated on TSA (tryptic soy agar). Plates were incubated (37 °C, 24 h), and STEC counts were enumerated. This resulted in a detection limit of 2.0 log CFU/g.

### 2.6. Flour Quality Evaluation

#### 2.6.1. Tempering Procedure

Non-sterile wheat (2.0 kg/treatment, three replications) was tempered at 1.25 and 1.5% SBS (wheat basis). The SBS concentrations used were selected based on the observed *E. coli* O121 and O26 reductions from the tempering experiments (≥3 log reductions). Tempering treatment of 0% SBS (water only) was maintained as a control. The SBS tempering solutions (33.7 and 40.5% *w*/*v* SBS) were prepared by dissolving the required amounts of SBS (grams) in corresponding amounts of distilled water. The required amount of tempering solution (160 mL) for tempering wheat to 17% moisture was calculated based on the initial wheat moisture content (10.3%). Wetted wheat kernels were mixed (30 min) using rotary mixing bins. For the SBS tempering treatments (1.25 and 1.5% SBS), water was poured first (80 mL) into the wheats followed by mixing (15 min) before the application of the prepared SBS solutions (80 mL) (33.7% *w*/*v*–1.25% SBS: 40.5% *w*/*v*–1.5% SBS). Wheat kernels were mixed for another 15 min after the addition of SBS treatments. For the control treatment (0% SBS), 160 mL of water (tempering solution) was poured in the wheat and mixed for 30 min. Wetted kernels were placed in resealable bags and tempered (24 h) prior to milling.

#### 2.6.2. Experimental Milling

Tempered wheats were milled using a Chopin LabMill (Chopin, France) with six milling steps (two break, one sizing, and three reduction steps). The feed rate was set at 5 g/s for the 1st break roll and 2.5 g/s for the subsequent milling steps. The laboratory-scale mill was brushed and vacuum-cleaned before milling each sample. The flour fractions obtained (break, sizing, and reduction flours) were mixed manually to make the straight grade flours for evaluation. The yields (%) of the mill fractions were expressed on an as-is basis and calculated as

yield (%) = (weight of milling fraction)/(weight of wheat) × 100(2)

#### 2.6.3. Particle Size Analysis

The particle size distributions (PSD) of the wheat flours were established by vacuum sieving using Hosokawa Alpine Jet Siever (Hosokawa, Augsberg, Germany); 100 g of flour was sieved through increasing sieve sizes ranging from 20 to 250 µm according to ASTM sieve standard sizes. The flour quantity (g) passing through the sieves was used for PSD analysis.

#### 2.6.4. Proximate Analysis

Moisture contents (% wet basis) of straight-grade flour and wheat were measured according to ASAE 352.2 [13]. The nitrogen content was measured according to American Association of Cereal Chemists (AACC) method 46-30.01 [14] with protein expressed as N × 5.7 for wheat or wheat flour. Ash was measured according to AOAC 923.03 [15]. The fat and fiber contents were measured based on AOAC 922.06 and AOAC 962.09 methods, respectively [15]. The carbohydrate content was determined based on calculation [100 − (%ash + %moisture + %fat + %protein)]. Measurements were expressed on an as-is (%) basis unless stated.

#### 2.6.5. Total Starch, Damaged Starch, and Falling Number

The total starch (%) content of the wheat flours obtained was measured according to AACC method 76-13.01 [14]. Damaged starch (UCD) was measured according to AACC method 76-33.01 [15] using an SDMatic^TM^ (Chopin, France). Falling number (s) values were measured according to AACC method 56-81.04 [14] using a Foss Alphatec^TM^ FN_o_ (Foss, Eden Prairie, MN, USA).

#### 2.6.6. pH Analysis

The pH measurement was conducted using a calibrated pH meter (Mettler Toledo, Columbus, OH, USA) with a combined electrode pH probe (LE438, Mettler Toledo, Columbus, OH, USA). Wheat pH was taken at the start (0.5 h) and end (24 h) of tempering. For wheat kernels, wheat flours, and milling fractions, pH was measured by making a 1:9 suspension of sample and distilled water. The suspensions were mixed for 15 min using a magnetic stirrer and rested for 10 min. The supernatant was decanted, and its pH was measured. For the tempering solutions, an aliquot of the freshly prepared solution was taken for analysis.

#### 2.6.7. Solvent Retention Capacity (SRC)

The SRC tests for the wheat flours were conducted based on AACC method 56-11.02 [14]. The SRC values of the flour were tested against the following solvents: deionized water, 5% lactic acid, 50% sucrose, and 5% sodium carbonate.

#### 2.6.8. Gluten Properties

Gluten characteristics of the wheat flours were measured according to AACC method 38-12.02 [14] using a Glutomatic apparatus (Perten Instruments, Springfield, IL, USA). Gluten characteristics (%) such as wet gluten, dry gluten, gluten index, and water binding capacity were calculated.

#### 2.6.9. Pasting Properties

The pasting properties of the wheat flours were measured using a Rapid Visco Analyzer (RVA) Model-4 (Newport Scientific, Warriewood, NSW, Australia). The test was conducted according to STD2 of AACC method 76-21.02 [14].

#### 2.6.10. Dough Rheology

Dough rheology tests were conducted using a MixoLab (Chopin Technologies, Villeneuve-la-Garenne, France) according to AACC method 54-60.01 [14]. The water absorption (%) of the flour samples was optimized prior to testing by adjusting absorption until the C1 value was within 1.10 ± 0.05 Nm after 8 min of mixing. Once this criterion was met, the test was allowed to proceed under the Chopin + protocol in 14% moisture basis.

#### 2.6.11. Flour Color Analysis

Color analysis of the wheat flours obtained was performed using a MiniScan EZ 4500 Colorimeter (HunterLab, Reston, VA, USA). Flours were placed in a sample cell holder, and L * (−black to +white), a * (−green to +red), and b * (−blue to +yellow) color values were measured.

#### 2.6.12. Bake Test

A bake test was conducted according to the formulation in AACC method 10-10.03 [14] consisting of (% flour basis) 100% flour, 5.3% yeast, 6.0% sugar, 1.5% salt, 3.0% shortening, and water (% based on Mixolab absorption). Doughs were made by mixing the ingredients in a 100 g-pin mixer (National Manufacturing Co., Lincoln, NE, USA) for 5 min. Doughs were rounded and fermented (26–27 °C, 90% RH, 30 min) before sheeting and molding. Doughs were placed in bake pans and proofed (26–27 °C, 90% RH, 60 min). Baking (204 °C, 24 min) was conducted in a reel-type oven. Loaves were cooled before bread analysis. Two replicate loaves were made for each tempering treatment.

#### 2.6.13. Bread Analysis

Loaves were cooled, and bread volume was measured by rapeseed displacement (AACC, 2010). The apparatus was calibrated with a wooden block (500 cc) before analysis. Loaf weight was measured and used for calculating specific bread volume (cc/g). Loaves were sliced (~15 mm thickness) using an electric slicer (Black &Decker, Towson, MD, USA) in preparation for c-cell and texture analyses. Slices were stored in polyethylene bags at ambient temperature (22–25 °C) before further analyses.

For c-cell analysis, two center slices (four slices/ treatment) were analyzed using the C-cell image analyzer (CCFRA Tech Ltd., Warrington, UK) with c-cell software 2.0. Images were taken of cell wall thickness and cell volume, and number measurements were taken.

Texture profile analysis was conducted using a TA. XT texture analyzer (Stable Microsystems, Godalming, UK) with a 2 in. diameter cylindrical probe. Each bread slice (eight slices/treatment) was analyzed with the following test parameters: 1.0 mm/s pre-test, 1.0 mm/s test, 5.0 mm/s posttest speed, 50% strain, and 20 g trigger force.

#### 2.6.14. Data Analysis

The tempering experiments followed a 7 × 6 factorial design with seven treatments (positive, negative, 0.5, 0.75, 1.0, 1.25, and 1.5% SBS) and six sampling intervals (0.5, 2, 6, 12, 18, and 24 h) for each STEC strain (O121 and O26). Mean log reductions for each treatment were analyzed using the GLIMMIX procedure with mean comparisons done using Tukey’s test (*p* ≤ 0.05). Three experimental replications of the tempering experiments were conducted.

The flour quality evaluation tests followed a completely randomized design (CRD) with three treatments (0, 1.25, and 1.5% SBS). Flour quality tests were conducted in duplicate (two wheat flour samples/ treatment) with values expressed as the mean (sd). Mean values were analyzed using the GLM procedure with comparisons done using Tukey’s test (*p* ≤ 0.05). All statistical analyses were conducted using SAS statistical software 9.3 (SAS Institute, Cary, NC, USA).

## 3. Results

### 3.1. E. coli O121 and O26 Load Reduction in Wheat during Tempering

Sodium bisulfate (pKa = 1.99, MW = 120.0 g/mol) is a dry acid salt that is soluble in water, dissociating into sodium, hydrogen, and sulfate ions [16]. The MIC of SBS against both *E. coli* O121 and O26 was 0.32% *w*/*v*. This demonstrates the inhibitory effects of SBS against STECs (O121 and O26), which is linked to its acidifying properties that lowered the pH of the growth media. *E. coli* O121 and O26 were used in this study as these serotypes were the most frequent cause of flour recalls [8].

**Figure 1:**
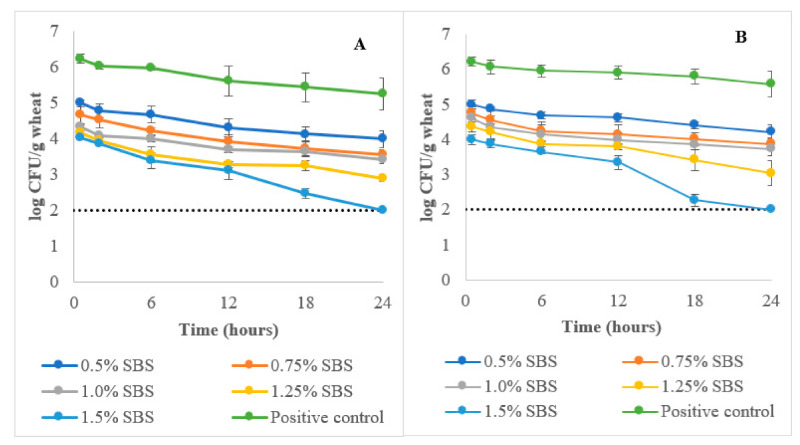
*E. coli* O121 (**A**) and O26 (**B**) load reductions in wheat during tempering; control corresponds to 0% SBS tempering treatment; dashed line corresponds to the limit of detection (2.0 log CFU/g wheat).

Based on Figure 1, the positive control showed a gradual decrease in *E. coli* (O121 and O26) load of the wheat during tempering. At the end of tempering (24 h) phase, there were small but significant (*p* ≤ 0.05) reductions in the *E. coli* O121 (0.8 logs) and O26 (0.6 logs) load of the wheat tempered with the control treatment (0% SBS). These reductions were based on the initial (0.5 h) wheat *E. coli* (O121 and O26) load (6.2 ± 0.3 log CFU/g). The observations demonstrate that the increased moisture levels of the wheat due to tempering were still insufficient to promote *E. coli* survival in wheat kernels. The tempering moisture used (17% moisture) in this study corresponds to wheat water activity (aw) levels of approximately 0.70 [17]. This a_w_ level is known to inhibit multiplication of bacterial cells such as *E. coli*, which requires a_w_ levels of ≥ 0.95 for growth. No colonies were recovered after plating in all sampling intervals for the negative control treatment.

The addition of SBS in the tempering water was effective in reducing the *E. coli* load of wheat (Figure 1). At 0.5% SBS, a ~1.0 log reduction was observed in 0.5 h of tempering. A maximum of a 2.0 log reduction for both *E. coli* serogroups was observed at 0.5 h tempering at SBS concentrations of 1.25 and 1.5% SBS. At the end of the tempering step, the 1.5% SBS concentration was able to reduce both *E. coli* O121 and O26 loads of wheat below detection limits (<2.0 log CFU/g) (*p* ≤ 0.05). Load reductions (log CFU/g) observed after tempering were 2.2 (0.5%), 2.5 (0.75%), 2.7 (1.0%), and 3.5 (1.25%) for *E. coli* O121 (*p* ≤ 0.05). As for *E. coli* O26, load reductions (log CFU/g) observed were 2.0 (0.5%), 2.4 (0.75%), 2.5 (1.0%), and 3.2 (1.25%) at the end of tempering (*p* ≤ 0.05). The reductions were calculated based on the *E. coli* (O121 and O26) load of the positive control sample at 0.5 h tempering. An interaction between tempering time and SBS concentrations was also observed (*p* ≤ 0.05) wherein longer tempering times were needed to achieve maximum *E. coli* (O121 and O26) load reduction in wheat due to SBS tempering.

SBS is recognized as a GRAS (Generally Recognized as Safe) substance. This classification means that it can be used by food manufacturers without the need for a pre-market review verifying its safety. Furthermore, the use of GRAS substances is permitted as long as they are used in accordance with the manufacturers’ Good Manufacturing Practices (GMPs). The observed MIC of SBS (0.32% *w*/*v*) in this study was also comparable with several common organic acids such as lactic (0.5%), and acetic (0.5%) [18,19]. The lower MIC observed indicates that lower amounts of SBS are required to inhibit STECs (O121 and O26), which could represent an economic benefit to manufacturers. 

Sodium bisulfate is also used for farm litter management [20]. Its acidifying property controls the enteric microbial load of animal manure with reported 2 to 5 log reductions upon application of SBS [21]. Table 1 shows that the addition of SBS lowered (*p* ≤ 0.05) the pH of the tempering water, resulting in a lower pH of the wheat kernels. These lowered pH values contributed to the reduction of the *E. coli* load of wheat during tempering. Higher pH values were observed for the treated wheats compared to the SBS tempering solutions prepared, showing that wheat kernels have some buffering capabilities allowing them to resist acidification [22]. In addition, the pH drops of wheat kernels decreased as the SBS tempering concentration increased, but higher STEC load reductions were still observed. The reductions could be explained by the increased acidity and osmolarity caused by the increased SBS concentration in the tempering solutions. This was also enhanced by the hygroscopic properties of SBS, which could have resulted in a drier wheat surface, resulting in the higher log reductions observed. The antimicrobial activity of acids is usually linked to the movement of acids across the cell membrane lowering internal cell pH, which disrupts cell activities [23,24]. As SBS is considered an acid salt, the ions from SBS salt changes the osmolarity of the wheat grain (cell environment) leading to cell dehydration. Bacterial cells expel water from their cells to balance out solute concentrations between the cell and its environment [25].

The maximum reductions observed upon addition of SBS were comparable with non-thermal intervention steps applied for wheats such as lactic acid (1.6 logs-non-O157 STECs, 1.8 logs-O157 STECs), ozone (3.2 logs-aerobic counts), and gamma irradiation (2 logs-aerobic counts) treatment of wheat [3,26,27]. SBS reductions were also comparable with the reductions from the thermal treatment of wheat flour (70 °C, 60 min) which gave 4.1 log reductions in *E. coli* O26 [5]. The results indicate that SBS tempering is effective in reducing the STEC load of wheat after tempering as normal levels of *E. coli* in wheat were reported to be only around 1.0 log CFU/g [28]. This reduction could then limit *E. coli* cross-contamination from wheat to milling equipment and mill fractions.

### 3.2. Flour Quality Evaluation

The impact of SBS tempering concentrations of 1.25 and 1.5% SBS on wheat flour quality was evaluated as they resulted in ≥3.0 log reductions after tempering. This reduction level is commonly accepted by most flour industry consumers for antimicrobial interventions applied to wheat flours [29]. No mandatory performance standards have been set by regulating bodies (e.g., Food and Drug Administration) with regards to pathogen reduction processes for wheat milling.

Table 2 summarizes the milling yields (% as-is) of wheat tempered with different SBS concentrations (0. 1.25, and 1.5% SBS). The wheat moisture after tempering (16.10–16.39%) for all treatments was within the moisture levels (15–17% moisture) used in hard wheat milling [30]. Bran yields decreased with increasing SBS concentrations while increasing (*p* ≤ 0.05) yields were observed for the shorts fraction. This suggests that SBS tempering could have increased bran friability resulting in more fine bran particles. Similar yields were observed (*p* > 0.05) for the straight-grade flours (73.66–74.72%). For the individual flour fractions, SBS tempering resulted in slightly higher sizing (SZ) flour and lower break (BK) flour yields while reduction (RD) flour yields were similar. Quantifying the yields for the individual flour fractions (break, sizing, and reduction) is important as they possess different attributes such as size and damaged starch content, altering the straight grade flour characteristics.

**Figure 2:**
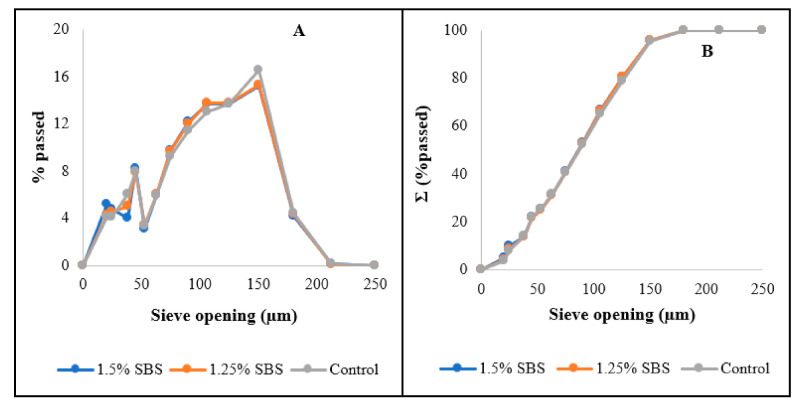
Particle size distribution (**A**) and cumulative particle size distribution (**B**) of wheat flours; control corresponds to 0% SBS tempering treatment.

The flours obtained also had relatively similar particle size distributions (Figure 2) wherein most of the flour particles had particle sizes ranging from 50 to 150 µm, satisfying the regulatory requirement for wheat flour particle size, which states that ≥98% of particles should be below 212 µm [31]. Aside from the regulatory requirements, particle size of wheat flours is also important since it affects the hydration and mixing properties of wheat flours and based on Figure 2, changes in the flour characteristics due to particle size for the treatments would be minimal as all treatments had relatively similar PSD.

Tempering with SBS also resulted in more acidic milling fractions (Table 3) with the 1.5% SBS treatment yielding the most acidic milling fractions (*p* ≤ 0.05). The pH values for the flour fractions (BK, SZ, RD, and SG flours) ranged from 5.23 to 5.56. These values were lower than the pH of the control treatment (pH = 6.67). These observations indicate that part of the SBS salt added to the wheat kernels diffused from the bran layer into the endosperm during tempering. The lower pH of the bran and shorts fraction indicate that more SBS molecules were retained on the surface of the wheat kernels (bran) relative to the endosperm layer.

The proximate composition of the wheat flours obtained from SBS-tempered wheats was comparable with that of the control treatment (Table 4). Moisture values for all the flours obtained were higher than the standard (14%) moisture for flours. This is related to the reduced workflow of laboratory-scale mills as moisture loss in flours during milling occurs due to the heat produced by successive roller milling. Higher ash and fiber contents (*p* ≤ 0.05) were observed with increasing SBS concentrations. This could be due to the increased concentration of salts in the wheat flours due to the added SBS salts in the tempering step. Higher (*p* ≤ 0.05) wheat flour protein and carbohydrate contents were observed with increasing SBS tempering concentrations although the changes were relatively small (<0.3%). As for the starch contents, tempering with SBS (1.25 and 1.50%) resulted in higher total and damaged starch contents, which could have implications for the pasting and dough properties of the wheat flours. Falling number (FN) values for all wheat flour samples were >250 s, indicating minimal amylase activity. Furthermore, FN values decreased with increasing SBS concentrations, which is related to the increased hydrolysis of the starch molecules into smaller glucose units due to acid and heat stresses [32]. The wheat flours from SBS-tempered wheats generally had lower L* and higher a* and b* values, indicating lower brightness, higher red, and yellow tones. The color changes could be linked to the higher ash and fiber contents of the 1.25 and 1.5% SBS wheat flours, which are indicators of bran contamination in wheat flours.

No significant differences (*p* > 0.05) were observed for the gluten index and dry gluten values (Table 5) of the wheat flours obtained. The observations indicate that SBS tempering did not significantly affect gluten strength (gluten index) and quantity (dry gluten). Lower (*p* ≤ 0.05) wet gluten values were observed for SBS-tempered wheats (1.25 and 1.5%) relative to the control, showing lower gluten water-binding capacity (Table 5). The differences were due to the lowered wheat flour pH, which could have altered the gluten formation and structure, reducing the flour water -binding capacity [33].

Solvent retention capacity (SRC) values are commonly used for assessing the baking performance of flours. The use of lactic acid, sodium carbonate, and sucrose solvents provide insights into the glutenin, damaged starch, pentosan, and gliadin quality, respectively, of wheat flours [34]. Water is a universal solvent that measures the overall quality of the flour [35]. The inclusion of SBS (1.25 and 1.5%) during wheat tempering yielded higher water, sucrose, and sodium carbonate SRC values for the wheat flours (Table 5). Lower lactic acid SRC values were observed for SBS wheat flours relative to the control. These observations indicate improved pentosan, damaged starch, gliadin characteristics, and lower gluten strength for the wheat flours produced from SBS-tempered wheats (1.25 and 1.50% SBS).

Higher peak viscosities and breakdown values (*p* ≤ 0.05) were observed (Table 5) for flours from SBS- tempered wheats. The higher peak viscosity observed could be due to the increased salt concentration in the flour due to the added SBS. The peak viscosity (RVA profile) of wheat flour suspensions was reported to increase with the addition of phosphate salts [36]. The higher breakdown and lower trough values indicate lowered hot paste holding strength. These changes could be explained by the increased rate of hydrolysis of the leeched amylopectin and amylose components due to heat and acid stresses [32]. Lower final viscosity, and setback values were observed for wheat flours from SBS wheat flours showing better retrogradation properties. The lower extent of retrogradation could be explained by the acidic pH and increased salt concentration present in the flour as these are known methods for retarding starch retrogradation during cooling [37]. Pasting temperatures for the wheat flours were similar, although slightly lower peak times were observed with increasing SBS tempering concentrations.

Similar values (*p* > 0.05) were observed for the water absorption, development time (time to C1), mixing stability, and amplitude (dough elasticity) values of SBS-wheat flours relative to the control (Table 6). C2 values for the wheat flours were also similar (*p* > 0.05), indicating similar protein qualities of wheat flours for all tempering treatments. C3 and C4 values for the 1.5% SBS were lower (*p* ≤ 0.05) than those of the control (0% SBS), indicating lower dough viscosity and gel stability during heating. C5 values of wheat flours from SBS-tempered wheats (1.25 and 1.5% SBS) were significantly lower than those of the control, indicating less starch retrogradation during the cooling phase. Mixolab behavior of the flours during the heating phase were relatively similar to the RVA curves, indicating that the differences could also be explained by the difference in salt and acidity of the wheat flours.

**Figure 3:**
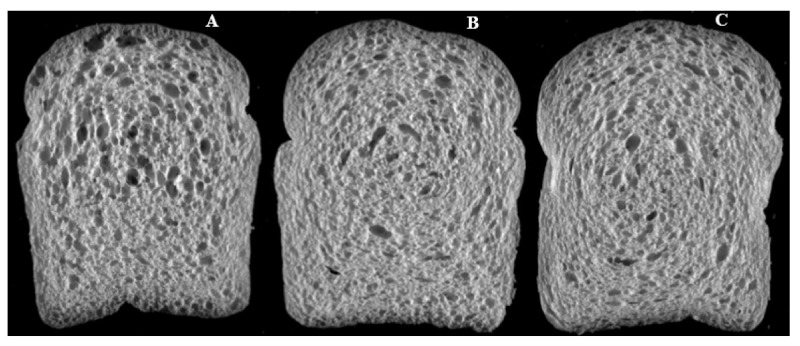
C-cell image of the loaf bread slices. Control–0% SBS (**A**), 1.25% SBS (**B**), and 1.50% SBS (**C**).

The breadmaking characteristics of the wheat flours are shown in Table 7 and the images of bread loaf bread slices are presented in Figure 3. Loaf volume and specific bread volume of SBS wheat flours (1.25 and 1.5% SBS) were comparable (*p* > 0.05) to those of the control (0% SBS). These characteristics are mostly dependent on the protein (gluten quantity and quality) attributes of the wheat flour [38]. This agrees with the trends observed for wheat flour gluten properties (Table 5) and protein quantity (Table 1). This observation also agrees with the mixolab values (C2 and stability) as these values were reported to have a high correlation with bread making qualities of wheat cultivars [39].

The crumb structure of the bread slices made from the obtained wheat flours is summarized in Table 7. Crumb structures of breads form as the water and alcohols evaporate from the dough during baking, resulting in the formation of the cells. The gluten structure then sets, maintaining the bread structure. Wheat flours from SBS-tempered wheats (1.25 and 1.5% SBS) produced breads with significantly (*p* ≤ 0.05) higher cell counts and smaller crumb structure (lower cell diameter and volume) compared to the control. Breads made from SBS-tempered wheats also had lower (*p* ≤ 0.05) wall thickness compared to the control (0% SBS). The significance of c-cell characteristics depends on the target characteristics of bread that is produced. Bread texture measurements are also shown in Table 7. Bread hardness (Table 7) generally increased with increasing amounts of SBS used for tempering, with the 1.5% SBS treatment giving the highest bread hardness value among the treatments. Furthermore, the resilience (ability of bread to spring back to its original shape), springiness (elastic recovery of bread after the removal of compression), chewiness (rubbery texture of bread during chewing), and cohesion (resistance to deformation of breads) increased with higher SBS concentrations used for tempering. Overall, SBS tempering resulted in breads with a finer crumb structure and higher texture profile values. This could be due to the increased salt and acidity concentration of the bread due to the SBS. These factors are known to help improve the structure of breads as they improve gluten strength [40].

## 4. Conclusions

The addition of SBS during tempering lowered the pH of the wheat grains, resulting in reductions of the *E. coli* O121 and O26 load of wheat after tempering. SBS tempering yielded reductions ranging from 2.0 log (0.5% SBS) to >4.0 log (1.5% SBS) after 24 h of tempering. Furthermore, maximum reductions occurred after tempering (24 h), indicating longer tempering times were necessary to achieve better load reductions. As for its effects on flour quality, SBS tempering of wheat yielded more acidic wheat flours, affecting their milling and baking quality. SBS tempering lead to higher ash and fiber contents, darker color, lower hot paste strength, and lower retrogradation. Breadmaking properties of the SBS wheat flours were comparable to those of the control, i.e., comparable load volumes, finer crumb structure, and higher textural properties. Future work could be conducted on the sensory evaluation of wheat flour products milled from SBS-tempered wheats to assess product acceptability. Furthermore, future research could also be conducted on the effects of SBS tempering on soft wheat flours (e.g., cookie and cake flours) as the acidity brought about by SBS could have more immediate impacts on quality as these flours do not usually include acidulants while bread flours (hard wheat flours) are usually acidified to improve dough characteristics. Validation studies on the reductions in STEC load could be done using STEC strains with known dry environment resistances (e.g., using outbreak strains isolated from dry foods) or by including a dry adaptation phase of STECs in stored wheat grains before tempering. Validation studies should also be conducted, as wheat characteristics (e.g., organic load, chemical composition, and surface characteristics) could differ due to cultivar type and geographical location, which could influence the effectiveness of SBS in reducing the wheat pathogen load In summary, SBS tempering was a viable intervention step for wheat milling as it reduced wheat STEC contamination and produced wheat flours with comparable properties to those produced using the conventional (water only) wheat tempering process.

## Figures and Tables

**Table 1:** Mean (sd) pH values taken during the tempering experiments.

Treatment (% SBS)	pH (Mean ± Standard Deviation)		
	pH ^1^	pH ^2^	pH ^3^
0	6.93 (0.15) ^a^	6.87 (0.06) ^a^	6.96 (0.12) ^a^
0.5	1.28 (0.04) ^b^	3.64 (0.04) ^b^	3.62 (0.04) ^b^
0.75	1.18 (0.02) ^c^	3.46 (0.08) ^cd^	3.46 (0.06) ^c^
1	1.10 (0.01) ^d^	3.29 (0.02) ^de^	3.25 (0.02) ^d^
1.25	1.02 (0.02) ^d^	3.20 (0.01) ^e^	3.20 (0.01) ^d^
1.5	0.85 (0.01) ^e^	3.11 (0.01) ^e^	3.13 (0.02) ^d^

Mean values for each column with different superscripts are significantly different (*p* ≤ 0.05) due to treatment; pH ^1^ corresponds to the pH of the tempering solution, pH ^2^ corresponds to the pH of wheat at the start of tempering (30 min), and pH ^3^ corresponds to the pH of wheat at the end of the tempering step (24 h).

**Table 2:** Mean (sd) milling yields (%) of tempered wheats.

Milling Fraction	Treatments (% SBS)		
	0	1.25	1.5
Bran	21.16 (0.57) ^a^	20.06 (0.30) ^b^	18.92 (0.21) ^c^
Shorts	5.17 (0.147) ^c^	5.91 (0.09) ^b^	6.36 (0.20) ^a^
BK Flour	27.86 (0.21) ^a^	26.50 (0.11) ^b^	26.35 (0.30) ^b^
SZ Flour	14.01 (0.15) ^b^	16.51 (0.19) ^a^	17.18 (0.11) ^a^
RD Flour	31.80 (0.67) ^a^	31.01 (0.50) ^a^	31.18 (0.27) ^a^
SG Flour (BK + SZ + RD)	73.66 (0.62) ^a^	74.02 (0.50) ^a^	74.72 (0.44) ^a^

Mean values in each column with different superscripts are significantly different due to treatment (*p* ≤ 0.05); BK–break, SZ–sizing, RD-reduction, and SG-straight- grade.

**Table 3:** Mean (sd) pH values for the wheat milling process.

Test Variable	Treatment (% SBS)		
	0	1.25	1.5
Tempering solution	6.25 (0.02) ^a^	1.52 (0.12) ^b^	1.05 (0.05) ^c^
Wheat ^1^	6.65 (0.04) ^a^	3.12 (0.02) ^b^	3.00 (0.02) ^c^
Wheat ^2^	6.59 (0.18) ^a^	3.11 (0.02) ^b^	3.00 (0.03) ^b^
Bran	7.36 (0.04) ^a^	4.05 (0.16) ^b^	3.87 (0.06) ^b^
Fine Bran	7.40 (0.01) ^a^	4.28 (0.03) ^b^	4.08 (0.06) ^b^
Shorts	7.31 (0.17) ^a^	4.77 (0.06) ^b^	4.61 (0.08) ^c^
BK Flour	6.74 (0.03) ^a^	5.44 (0.01) ^b^	5.23 (0.03) ^c^
SZ Flour	6.51 (0.01) ^a^	5.75 (0.02) ^b^	5.56 (0.01) ^c^
RD Flour	6.59 (0.03) ^a^	5.56 (0.04) ^b^	5.32 (0.01) ^c^
SG Flour	6.67 (0.05) ^a^	5.60 (0.04) ^b^	5.41 (0.01) ^c^

Mean values in each row (test variable) with different superscripts are significantly different (*p* ≤ 0.05) due to treatment; wheat ^1^-pH measured 30 min after application of tempering solutions, wheat ^2^-pH measured after tempering (24 h); BK–break, SZ–sizing, RD-reduction, and SG-straight-grade.

**Table 4:** Mean (sd) physico-chemical characteristics of wheat flours.

Test Variable	Treatment (% SBS)		
	0	1.25	1.5
Moisture (%wet basis)	15.57 (0.13) ^a^	15.02 (0.09) ^b^	14.99 (0.05) ^c^
Ash (%)	0.44 (0.03) ^c^	0.62 (0.01) ^b^	0.71 (0.01) ^a^
Protein (%)	10.81 (0.03) ^c^	10.94 (0.03) ^ab^	10.92 (0.09) ^bc^
Fat (%)	0.54 (0.02) ^b^	0.48 (0.01) ^b^	0.65 (0.02) ^a^
Fiber (%)	0.18 (0.04) ^c^	0.25 (0.01) ^bc^	0.37 (0.04) ^ab^
Carbohydrates (%)	72.59 (0.07) ^c^	72.88 (0.05) ^ab^	72.68 (0.04) ^bc^
Total Starch (%)	66.28 (1.42) ^c^	71.27 (0.38) ^ab^	68.74 (0.95) ^bc^
Damaged Starch (UCD)	14.63 (0.51) ^b^	14.73 (0.70) ^b^	16.57 (0.45) ^a^
Falling Number (sec)	587.3 (15.6) ^a^	522.0 (12.7) ^b^	517.3 (30.4) ^b^
L* (0-black to 100-white)	89.21 (0.13) ^a^	89.76 (0.42) ^a^	88.45 (0.43) ^b^
a* (−green to +red)	0.58 (0.04) ^b^	0.92 (0.02) ^a^	0.93 (0.02) ^a^
b* (−blue to +yellow)	9.70 (0.17) ^b^	10.17 (0.05) ^a^	10.25 (0.06) ^a^

Mean values in each row (test variable) with different superscripts are significantly different (*p* ≤ 0.05) due to treatment.

**Table 5:** Mean (sd) gluten, solvent retention capacity, and RVA characteristics of wheat flours.

Test Variable	Treatment (% SBS)		
	0	1.25	1.5
Gluten properties (%)			
Wet gluten	26.17 (0.43) ^a^	26.38 (0.29) ^a^	24.66 (0.28) ^b^
Dry gluten	9.12 (0.14) ^a^	9.75 (0.50) ^a^	9.10 (0.42) ^a^
Gluten Index	99.42 (0.28) ^a^	99.04 (0.26) ^a^	99.80 (0.28) ^a^
Water-binding capacity	17.17 (0.43) ^ab^	16.64 (0.21) ^b^	15.56 (0.14) ^c^
Solvent retention capacity (%)			
Water	54.60 (0.86) ^b^	57.01 (0.61) ^a^	58.53 (0.34) ^a^
50% sucrose	73.40 (0.67) ^b^	75.68 (0.39) ^a^	75.49 (0.86) ^a^
5% lactic acid	106.22 (0.35) ^a^	84.52 (0.40) ^b^	80.29 (0.41) ^b^
5% sodium carbonate	62.95 (0.45) ^c^	66.22 (0.59) ^b^	67.71 (0.23) ^a^
Rapid visco analyzer (RVA) characteristics			
Peak viscosity (RVU)	3936.3 (83.7) ^b^	4304.0 (59.5) ^a^	4288.7 (34.2) ^a^
Trough (RVU)	2005.3 (25.1) ^b^	2068.0 (34.9) ^ab^	1966.0 (7.5) ^c^
Breakdown (RVU)	1931.0 (86.5) ^b^	2236.0 (27.5) ^a^	2322.7 (27.8) ^a^
Final viscosity (RVU)	4902.0 (85.3) ^a^	4735.7 (62.6) ^b^	4561.7 (24.0) ^c^
Setback (RVU)	2896.7 (82.3) ^a^	2667.3 (30.5) ^b^	2595.7 (20.8) ^b^
Peak time (mins)	8.98 (0.10) ^a^	8.94 (0.03) ^ab^	8.87 (0.04) ^b^
Pasting temperature (°C)	65.3 (0.20) ^a^	64.8 (0.80) ^a^	64.2 (1.30) ^a^

Mean values in each row (test variable) with different superscripts are significantly different (*p* ≤ 0.05) due to treatment; solvent retention capacity values are expressed on a 14% moisture basis; breakdown was calculated as the difference between the peak viscosity and trough, setback was calculated as the difference between the final viscosity and trough.

**Table 6:** Mean (sd) mixolab characteristics of wheat flours.

Test Variable	Treatment (% SBS)		
	0	1.25	1.5
Water absorption (%)	54.5 (0.24) ^a^	54.4 (0.29) ^a^	54.7 (0.13) ^a^
Development time (min)	2.21 (0.26) ^a^	1.90 (0.24) ^a^	2.00 (0.19) ^a^
Stability (min)	8.90 (0.20) ^a^	9.30 (0.21) ^a^	8.98 (0.22) ^a^
Amplitude (Nm)	0.06 (0.01) ^a^	0.07 (0.01) ^a^	0.08 (0.01) ^a^
C1 (Nm)	1.10 (0.03)	1.10 (0.04)	1.10 (0.03)
CS (Nm)	1.05 (0.02) ^a^	1.02 (0.02) ^a^	1.04 (0.02) ^a^
C2 (Nm)	0.47 (0.01) ^a^	0.46 (0.01) ^a^	0.46 (0.01) ^a^
C3 (Nm)	2.17 (0.02) ^a^	2.16 (0.02) ^a^	2.11 (0.03) ^b^
C4 (Nm)	2.03 (0.03) ^a^	2.01 (0.02) ^a^	1.94 (0.04) ^b^
C5 (Nm)	3.28 (0.07) ^a^	3.12 (0.04) ^b^	3.07 (0.02) ^b^

Mean values in each row that have different superscripts are significantly different (*p* ≤ 0.05) due to treatment; Mixolab tests were conducted using the “Chopin +” protocol on a 14% moisture basis.

**Table 7:** Mean (SD) baking and bread characteristics of wheat flours.

Test Variable	Treatment (% SBS)		
	0	1.25	1.5
Bread Characteristics			
Bread volume (cc)	545.0 (7.1) ^a^	562.5 (10.6) ^a^	542.5 (3.5) ^a^
Specific bread volume (cc/g)	3.90 (0.82) ^a^	3.90 (0.08) ^a^	3.70 (0.04) ^a^
C cell analysis			
Number of cells (n)	2354.0 (167.8) ^b^	2990.5 (134.6) ^a^	2930.3 (132.2) ^a^
Cell diameter (mm)	2.48 (0.20) ^a^	2.00 (0.09) ^b^	1.77 (0.06) ^b^
Wall thickness (mm)	0.48 (0.02) ^a^	0.44 (0.01) ^b^	0.43 (0.01) ^b^
Cell volume (cc)	8.72 (0.79) ^a^	6.58 (0.43) ^b^	5.75 (0.25) ^b^
Texture Properties			
Hardness (N)	5.55 (0.60) ^c^	7.41 (0.74) ^b^	8.90 (1.54) ^a^
Resilience (%)	23.56 (1.96) ^b^	29.29 (2.02) ^a^	27.98 (1.83) ^a^
Cohesion	0.51 (0.02) ^b^	0.62 (0.06) ^a^	0.59 (0.02) ^a^
Springiness (%)	102.70 (5.22) ^a^	111.76 (14.49) ^a^	120.98 (24.26) ^a^
Chewiness (N)	2.92 (0.41) ^c^	5.11 (0.94) ^b^	6.54 (1.21) ^a^

Mean values in each row (test variable) that have different superscripts are significantly different (*p* ≤ 0.05) due to treatment.

## Data Availability

Data will be provided upon reasonable request by author Jared Rivera.

## References

[1] Aydin A., Paulsen P., Smulders F. (2009). The physico-chemical and microbiological properties of wheat flour in Thrace. Turk. Turk. J. Agric. For..

[2] Pascale M., Haidukowski M., Lattanzio V., Silvestri M., Ranieri R., Visconti A. (2011). Distribution of T-2 and HT-2 toxins in milling fractions of durum wheat. J. Food Prot..

[3] Sabillon L., Stratton J., Rose D., Binachini A. (2019). Effect of saline organic acid solutions applied during soft wheat tempering on microbial load and flour functionality. Cereal Chem..

[4] Finn S., Condell O., McClure P., Amezquita A., Fanning S. (2013). Mechanisms of survival, responses, and sources of Salmonella in low-moisture environments. Front. Microbiol..

[5] Forghani F., den Bakker M., Futral A., Diez-Gonzalez F. (2018). Long-term survival and thermal death kinetics of Enterohemorrhagic Escherichia coli serogroups O26, O103, O111, and O157. Appl. Environ. Microbiol..

[6] Neil K., Biggerstaff G., MacDonald J.K., Trees E., Medus C., Musser K., Stroika S., Zink D., Sotir M. (2012). A novel vehicle for transmission of Escherichia coli O157:H7 to humans: Multistate outbreak of E. coli O157:H7 infections associated with consumption of ready-to-bake commercial prepackaged cookie dough--United States, 2009. Clin. Infect. Dis..

[7] Food and Drug Administration Recalls, Market Withdrawals, and Safety Alerts. https://www.fda.gov/safety/recalls-market-withdrawals-safety-alerts.

[8] Centers for Disease Control and Prevention “Multistate Outbreak of Shiga Toxin-Producing Escherichia coli Infections Linked to Flour (Final Update)”. https://www.cdc.gov/ecoli/2016/o121-06-16/index.html.

[9] Los A., Ziuzina D., Akkermans S., Boehm D., Cullen P., Van Impe J., Bourke P. (2018). Improving microbiological safety and quality characteristics of wheat and barley by high voltage atmospheric cold plasma closed processing. Food Res. Int..

[10] Dhillon B., Wiesenborn D., Wolf-Hall C., Manthey F. (2009). Development and evaluation of an ozonate water system for antimicrobial treatment of durum wheat. J. Food Sci..

[11] Sabillon L., Stratton J., Rose D., Flores R., Bianchini A. (2016). Reduction in microbial load of wheat by tempering with organic acid and saline solutions. Cereal Chem..

[12] Clinical and Laboratory Standards Institute (2012). Performance Standards for Antimicrobial Susceptibility Testing.

[13] American Society of Agricultural Engineers International (1988). Method S352.2-Moisture Measurement-Unground Grain and Seeds.

[14] American Association of Cereal Chemists International (2010). Approved Methods of Analysis. Method 46-30.01, Crude Protein-Combustion Method; Method 76-13.01, Total Starch Assay Procedure; Method 76-33.01, Damaged Starch—Amperometric Method; Method 56-81.04, Determination of Falling Number; Method 56-11.02, Solvent Retention Capacity Profile; Method 38-12.02, Wet Gluten, Dry Gluten, Water-Binding Capacity, and Gluten Index Method 76-21.02, General Pasting Method for Wheat or Rye Flour or Starch Using the Rapid Visco Analyzer; Method 54-60.01, Determination of Rheological Behavior as a Function of Mixing and Temperature Increase in Wheat Flour and Whole Wheat Meal by Mixolab; Method 10-10.03, Optimized Straight Dough Bread-Making Method; Method 10-05.01, Guidelines for Measurement of Volume by Rapeseed Displacement.

[15] Association of Official Analytical Chemists International (2019). Official Methods of Analysis. Method 923.03—Ash Determination; Method 922.06-Fat in Flour Acid Hydrolysis Method; Method 962.09—Fiber in Animal Feed and Pet Food.

[16] Sun H., Pan Y., Zhao Y., Jackson W.A., Nuckels L.M., Malkina I.L., Arteage V.E., Mitloehner F.M. (2008). Effects of sodium bisulfate on alcohol, amine, and ammonia emissions from dairy slurry. J. Environ. Qual..

[17] Carter B., Morris C., Weaver G., Carter A. (2015). The Case for Water Activity as a Specification for Wheat Tempering and Flour Production. Cereal Foods World.

[18] Wali M.K., Abed M.M. (2019). Antibacterial activity of acetic acid against different types of bacteria causes food spoilage. J. Food Technol. Preserv..

[19] Wang C., Chang T., Yang H., Cui M. (2015). Antibacterial mechanism of lactic acid on physiological and morphological properties of Salmonella Enteritidis, Escherichia coli and Listeria monocytogenes. Food Control..

[20] Pope M.J., Cherry T.E. (2000). An evaluation of the presence of pathogens on broilers raised on poultry litter treatment-treated litter. Poult. Sci. J..

[21] McGarvey J.A., Stackhouse K.R., Miller W.G., Stanker L.H., Hnasko R., Mitloehner F. (2011). Effects of sodium bisulfate on the bacterial population structure of dairy cow waste. J. Appl. Microbiol..

[22] Nayeri M.D., MdTahir P., Harun J., Abdullah L.C., Bakar E.S., Jawaid M., Namvar F. (2013). Effects of temperature and time on the morphology, pH, and buffering capacity of bast and core kenaf fibers. Biol. Res..

[23] Brul S., Coote P. (1999). Preservative agents in foods—Mode of action and microbial resistance mechanisms. Int. J. Food Microbiol..

[24] Russell J.B. (1992). Another explanation for the toxicity of fermentation acids at low pH: Anion accumulation versus uncoupling. J. Appl. Microbiol..

[25] Davidson P.M., Taylor M.T., Doyle M.P., Beuchat L.R. (2006). Chemical Preservatives and natural Antimicrobial Compounds. Food Microbiology: Fundamentals and Frontiers.

[26] Sabillon L. (2018). A Risk-Based Approach to Evaluate the Impact of Interventions at Reducing the Risk of Foodborne Illness Associated with Wheat-Based Products. Ph.D. Thesis.

[27] Weaver G., Atkins-Lewenthal E., Allen B., Baker S., Hoerning D., Peterson A., Schumacher R., Warren B. (2011). Microbial Reduction in a Processing Stream of a Milled Product. U.S. Patent.

[28] Sperber W.H. (2007). Role of microbiological guidelines in the production and commercial use of milled cereal grains: A practical approach for the 21st century. J. Food Prot..

[29] Sterk R. Millers Await Advancements in Controlling Pathogens in Flour. https://www.foodbusinessnews.net/articles/10823-millers-await-advancements-in-controlling-pathogens-in-flour.

[30] Posner E.S., Hibbs A.N. (2005). Wheat Flour Milling.

[31] Codex Alimentarius-International Food Standards Standard for Wheat Flour. http://www.fao.org/fao-who-codexalimentarius/sh-proxy/en/?lnk=1&url=https%253A%252F%252Fworkspace.fao.org%252Fsites%252Fcodex%252FStandards%252FCXS%2B152–1985%252FCXS_152e.pdf.

[32] Hirashima M., Takahashi R., Nishinari K. (2005). Effects of adding acids before and after gelatinization on the viscoelasticity of cornstarch pastes. Food Hydrocoll..

[33] Garg S., Cran M., Mishra V. (2019). Effect of heating and acidic pH on characteristics of gluten suspension. Int. J. Food Sci. Tech..

[34] Ram S., Dawar V., Singh R., Shoran J. (2005). Application of solvent retention capacity test for the prediction of mixing properties of wheat flour. J. Cereal Sci..

[35] Guttieri M., Bowen D., Gannon D., O’Brien K., Souza E. (2001). Solvent retention capacities of irrigated soft white spring wheat flours. Crop Sci..

[36] Zhou Y., Hou G. (2012). Effects of phosphate salts on the pH values and Rapid Visco Analyser (RVA) pasting parameters of wheat flour suspensions. Cereal Chem..

[37] Wang S., Li C., Copeland L., Niu Q., Wang S. (2015). Starch Retrogradation: A comprehensive review. Compr. Rev. Food Sci. Food Saf..

[38] Collado-Fernandez M., Caballero B. (2003). Bread-Breadmaking Process. Encyclopedia of Food Sciences and Nutrition.

[39] Dhaka V., Guila N., Khatkar B.S. (2012). Application of Mixolab to Assess the Bread Making Quality of Wheat Varieties. OMICS Int..

[40] Nahar N., Madzuki I., Izzah N., Ab Karim M., Ghazali H., Karim R. (2019). Bakery Science of Bread and the Effect of Salt Reduction on Quality: A Review. Borneo J. Sci. Technol..

